# A quadruple dissociation of reward-related behaviour in mice across excitatory inputs to the nucleus accumbens shell

**DOI:** 10.1038/s42003-023-04429-6

**Published:** 2023-01-30

**Authors:** Erin B. Lind, Brian M. Sweis, Anders J. Asp, Manuel Esguerra, Keelia A. Silvis, A. David Redish, Mark J. Thomas

**Affiliations:** 1 Department of Neuroscience, University of Minnesota, 6-145 Jackson Hall, 321 Church St SE, Minneapolis, MN 55455 USA; 2 Medical Discovery Team on Addiction, University of Minnesota, 3-432 McGuire Translational Research Facility, 2001 6th St SE, Minneapolis, MN 55455 USA; 3 Department of Psychiatry, Friedman Brain Institute, Icahn School of Medicine at Mount Sinai, 1 Gustave L. Levy Pl, New York, NY 10029 USA; 4 Rehabilitation Medicine Research Center, Department of Physical Medicine and Rehabilitation, Mayo Clinic, 200 First St SW, Rochester, MN 55905 USA

**Keywords:** Neural circuits, Reward, Motivation

## Abstract

The nucleus accumbens shell (NAcSh) is critically important for reward valuations, yet it remains unclear how valuation information is integrated in this region to drive behaviour during reinforcement learning. Using an optogenetic spatial self-stimulation task in mice, here we show that contingent activation of different excitatory inputs to the NAcSh change expression of different reward-related behaviours. Our data indicate that medial prefrontal inputs support place preference via repeated actions, ventral hippocampal inputs consistently promote place preferences, basolateral amygdala inputs produce modest place preferences but as a byproduct of increased sensitivity to time investments, and paraventricular inputs reduce place preferences yet do not produce full avoidance behaviour. These findings suggest that each excitatory input provides distinct information to the NAcSh, and we propose that this reflects the reinforcement of different credit assignment functions. Our finding of a quadruple dissociation of NAcSh input-specific behaviours provides insights into how types of information carried by distinct inputs to the NAcSh could be integrated to help drive reinforcement learning and situationally appropriate behavioural responses.

## Abstract (web summary)

An optogenetic, spatial self-stimulation task in mice reveals how information carried by distinct inputs to the nucleus accumbens shell is potentially integrated to drive behavioural responses.

## Introduction

Goal-directed behaviour involves the integration of multiple cognitive, emotional, and motivational processes to coordinate the appropriate execution of environment- and situation-specific behaviours. The nucleus accumbens (NAc) is a key limbic-motor interface implicated in the integration of information which drives behavioural responses to motivationally relevant stimuli^1–4^. While both the shell (NAcSh) and core subregions are involved in reward valuation^5–9^, the NAcSh subregion is of particular interest as it receives input from both cortical and limbic brain regions and is well situated to receive, integrate, and respond to information about both appetitive and aversive stimuli^10,11^. Additionally, alterations in excitatory transmission in NAcSh have been implicated in both adaptive and maladaptive motivated behaviour, and maladaptive learning and experience-dependent plasticity at excitatory synapses within the NAcSh are thought to underlie a range of psychiatric disorders including depression, addiction, and schizophrenia^12–15^. Despite this, relatively little is known regarding how individual glutamatergic inputs to the NAcSh may guide reward valuation in goal-directed behaviour, or how these different inputs may contribute to the acquisition or expression of adaptive or maladaptive behaviours.

The NAcSh is primarily composed of medium spiny GABAergic neurons (MSNs) that receive excitatory input from multiple source nuclei including the medial prefrontal cortex (mPFC), ventral hippocampus (vHPC), basolateral amygdala (BLA), and paraventricular thalamus (PVT)^11,16,17^. These source nuclei are most often implicated in regulating emotional processing and the expression of approach/avoidance behaviour, although the direction of these effects is sometimes equivocal, particularly within the BLA and PVT^18–23^. Optogenetic approaches to selectively activate mPFC, vHPC, BLA, and PVT excitatory projections to the NAcSh during real time place preference or instrumental self-stimulation assays indicate each of these pathways are involved in emotional and motivational valence. Contingent stimulation of PVT → NAcSh inputs reduces or has variable effects on real time place preference^19,24,25^ but can also support lever-based self-stimulation^25^. In contrast, contingent stimulation of mPFC → NAcSh, vHPC → NAcSh, or BLA → NAcSh inputs consistently support both real time place preference and instrumental responding, indicating that stimulation of these inputs is rewarding^25–27^. Given the finding that mPFC → NAcSh, vHPC → NAcSh, and BLA → NAcSh inputs all have positive valence, it has been proposed that amount, rather than source, of excitatory drive to the NAcSh is what is most relevant for motivated behaviour^28^. However, given the diverse and often complex roles of the mPFC, vHPC, and BLA in affective, motivational, and cognitive function^29–33^, it is possible that input-specific differences in reward-directed behaviour were simply not detectable using classic paradigms, or that other aspects of motivated behaviour, such as reward valuation, reinforcement learning, and/or computational decision-making processes may be involved.

It remains unclear what, if any, specific information is provided by different excitatory inputs to the NAcSh or how this may regulate specific aspects of reward valuation or decision-making during foraging behaviour^9,34^. Thus, in the present study we utilized a spatially dependent optogenetic self-stimulation task to investigate strategies employed by mice to obtain or avoid that optogenetic stimulation. We found that self-stimulation of individual excitatory inputs to the NAcSh resulted in distinct behavioural patterns, which we propose relate to the reinforcement of different credit assignment functions.

## Results

### Approach

**Fig. 1:**
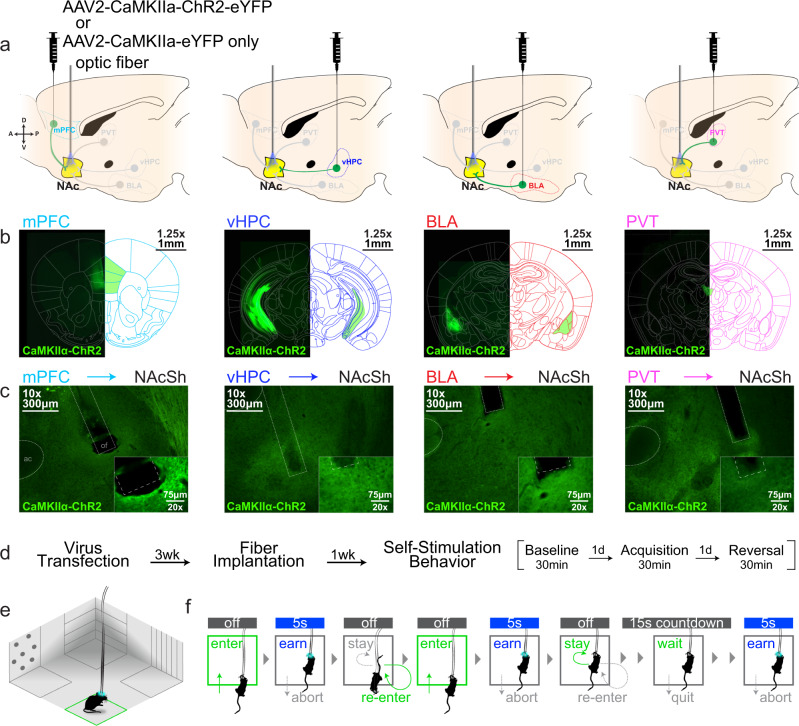
Neural pathway targets and optogenetic self-stimulation behaviour task. **a** Viral vector injections and optic fiber implants targeted specific excitatory inputs to the NAcSh. **b** Representative images (4x) from coronal slices (40 µm) showing viral localization and spread. **c** Representative images of input-specific ChR2+ terminal regions in NAcSh (10x, insets at 20x). The optical fiber (of) and anterior commissure (ac) are depicted with straight and rounded dashed lines, respectively. **d** Experimental timeline. Each session was 30 min long with one session per day. **e** Optogenetic stimulation was inactive during baseline, active in one zone during acquisition, and active in a different zone during reversal (30 Hz, 5 ms pulse width, 5 s max stimulation per bout). **f** Illustration of reward-related decisions during the task. A mouse could exit and re-enter the active zone to bypass a 15 s “timeout” period, wait in the active zone during the timeout for additional stimuli, or any combination of these two options.

We used optogenetic methods to selectively activate different excitatory inputs to the NAcSh (Fig. 1a, Supplementary Fig. 1a). An adeno-associated virus (AAV2) was used to express channelrhodopsin2 (ChR2) or control fluorophore (eYFP) under a Ca^2+^/calmodulin-dependent protein kinase IIa (CaMKIIa) promoter to drive eYFP or ChR2-eYFP expression in glutamatergic neurons in either the medial prefrontal cortex (mPFC), ventral hippocampus (vHPC), basolateral amygdala (BLA), or paraventricular thalamus (PVT) (Fig. 1b, Supplementary Fig. 1b). Optical fibers were then implanted bilaterally above eYFP+ or ChR2+ terminals in the NAcSh to allow for selective activation of mPFC → NAcSh, vHPC → NAcSh, BLA → NAcSh, or PVT → NAcSh pathways (Fig. 1c, Supplementary Fig. 1c). To identify potential input-specific differences in behavioural strategies exhibited during reinforcement learning, we assessed optogenetic self-stimulation behaviour in an open-field spatial task (Fig. 1d; Supplementary Fig. 2). The spatial arena used for this task consisted of an open square box (20'' x 20'') with four spatially restricted and contextually distinct zones, one in each corner (Fig. 1e). Mice were allowed to freely explore during an initial baseline session. Acquisition of self-stimulation behaviour was assessed the next day by pairing entry into one of these corner zones with optogenetic stimulation of either mPFC → NAcSh, vHPC → NAcSh, BLA → NAcSh, or PVT → NAcSh inputs. A reversal test was conducted the following day by switching the location of the active zone to confirm the reward valence of each input as well as the behavioural strategy that was observed during acquisition.

**Fig. 2:**
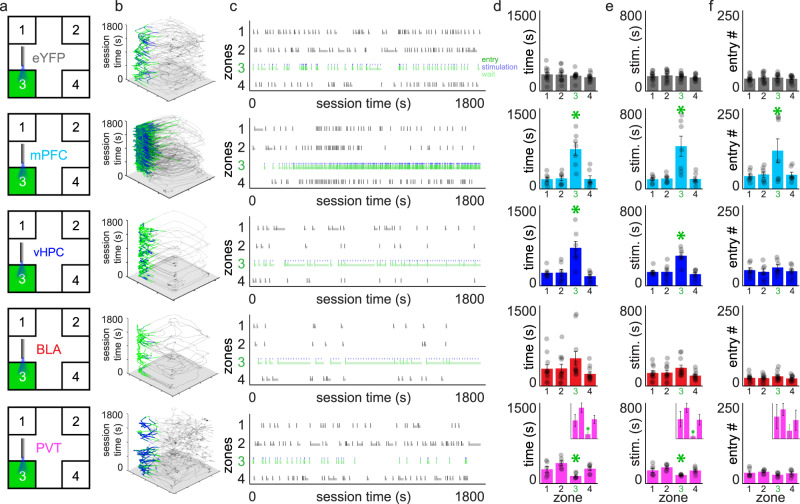
Self-stimulation of different NAcSh inputs results in input-specific behavioural strategies. Different NAcSh input groups are depicted by different colours throughout: eYFP controls (gray, *n* = 12), mPFC → NAcSh (cyan, *n* = 7 mice), vHPC → NAcSh (blue, *n* = 7 mice), BLA → NAcSh (red, *n* = 11 mice), and PVT → NAcSh (pink, *n* = 6 mice). **a** Mice explored the spatial arena during a 30 min acquisition session with one active (3; green) and three inactive (1,2,4) corner zones. **b** *x*–*y* position tracking for representative mice throughout the session (green = active zone position; blue = stimulation position). **c** Raster plots showing individual zone entries over time in representative mice (from b). Green ticks indicate active zone entries, blue ticks indicate stimulation onset, green shading indicates time spent waiting during the timeout in the active (full height shading) and inactive (half height shading) zones. **d** Total time spent across the four corner zones. Active-zone time was increased in mPFC → NAcSh and vHPC → NAcSh mice and decreased in PVT → NAcSh mice (inset presented for clarity). **e** Total stimulation (real or mock) received in each corner zone. Stimulation time was increased in mPFC → NAcSh and vHPC → NAcSh mice and decreased in PVT → NAcSh mice. **f** Total entries made into the different corner zones. Active-zone entries were only increased (vs. inactive zones) in mPFC → NAcSh mice. Despite the reduced stimulation received in PVT → NAcSh mice, these mice did not avoid entry into the active zone. Group data are expressed as mean ± SEM. Main zone effects within each group and metric were identified using one-way ANOVAs. *p < 0.05 active vs. all other inactive zones, Student’s post hoc comparisons.

**Fig. 3:**
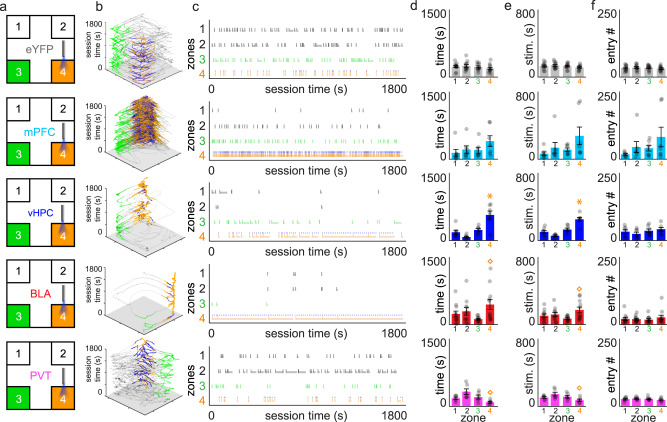
Expression of NAcSh input-specific behavioural strategies during reversal testing. Different NAcSh input groups are depicted by different colours throughout: eYFP controls (gray, *n* = 12 mice), mPFC → NAcSh (cyan, *n* = 7 mice), vHPC → NAcSh (blue, *n* = 7 mice), BLA → NAcSh (red, *n* = 11 mice), and PVT → NAcSh (pink, *n* = 6 mice). **a** Following acquisition, mice explored the testing arena with a new active zone (4; orange). Inactive zones included the previously active zone (3; green) and zones 1 and 2 (black). **b** *x*–*y* position tracking for representative mice throughout the session (orange = position in active zone; blue = position during stimulation; green = position in previously active zone). **c** Raster plots showing individual zone entries over time in representative mice (same mice as in b). Orange shading indicates time spent remaining in the active zone. **d** Total time spent across the four corner zones. **e** Total stimulation received (real or mock) received. **f** Total entries made into the different corners. Group data are expressed as mean ± SEM. Main zone effects within each group and metric were identified using one-way ANOVAs. ^◊^*p* < 0.05 main effect across zones, *p < 0.05 active vs. all other inactive zones, Student’s post-hoc tests.

To identify strategies the mice exhibited while engaging with stimulation-paired environments during testing, we employed stimulation parameters that were contingent on the mouse’s behaviour but also allowed mice flexibility in how they received optogenetic self-stimulation within the spatial arena. Entry into a stimulation-paired zone triggered a 465 nm blue-light LED for up to 5 s (30 Hz, 5 ms pulse width), followed by a 15 s non-reinforced “timeout” period. Mice could terminate stimulation early by exiting the active zone. If mice received the full 5 s stimulation, they could remain in the zone throughout the timeout period to gain additional bouts of stimulation without taking any further action. They could also bypass the timeout period by exiting and re-entering the zone to trigger another bout of stimulation. Mice could freely vary across these options throughout the test (Fig. 1f, Supplementary Fig. 2). Using this approach, we found that eYFP control mice explored the spatial arena similarly across transfection groups during the baseline, acquisition, and reversal sessions, suggesting that viral surgery and light delivery into the NAcSh alone did not impact behaviour in the spatial arena (Supplementary Fig. 3). Thus, data from eYFP mice was pooled for subsequent analysis (*n* = 12 mice). Initially, separate one-way ANOVAs were used to assess behaviour across the four corner zones during baseline (Supplementary Fig. 4), acquisition (Fig. 2a), and reversal testing (Fig. 3a). These analyses revealed that while eYFP and ChR2+ mice exhibited similar exploratory behaviour during baseline testing, stark behavioural differences emerged when mPFC (*n* = 7), vHPC (*n* = 7), BLA (*n* = 11), or PVT (*n* = 6) inputs to the NAcSh were selectively activated upon active zone entry during acquisition and reversal sessions.

### Expression of different behavioural strategies during acquisition in ChR2 + mice

During acquisition, ChR2 + , but not eYFP control mice, showed alterations in behaviour across the different corner zones during optogenetic stimulation; however, the type of alteration differed between the different ChR2+ input pathways (Fig. 2). For example, both mPFC → NAcSh (*F*_3,24_ = 14.7956, *p* < 0.0001, *ɳ*^2^ = 0.65) and vHPC → NAcSh (*F*_3,24_ = 11.4987, *p* < 0.0001, *ɳ*^2^ = 0.59) stimulation produced real-time place preferences and increased the time mice spent in stimulation-paired corners (Fig. 2d), resulting in a significant amount of optogenetic stimulation of these two inputs being self-administered (Fig. 2e, *F*_3,24_ = 9.7834, *p* = 0.0002, *ɳ*^2^ = 0.55 and *F*_3,24_ = 13.0889, *p* < 0.0001, *ɳ*^2^ = 0.62, respectively). In contrast, BLA → NAcSh mice showed only non-significant trends for elevated active zone-directed behaviour as assessed by these measures (Fig. 2d, *F*_3,40_ = 2.0105, *p* = 0.1279 and Fig. 2e, *F*_3,40_ = 2.1827, *p* = 0.1051). While PVT → NAcSh mice also showed altered time spent (Fig. 2d, *F*_3,20_ = 5.8616, *p* = 0.0048, *ɳ*^2^ = 0.47) and optogenetic stimulation received (Fig. 2e, *F*_3,20_ = 4.9177, *p* = 0.0102, *ɳ*^2^ = 0.42) during the acquisition session, active zone-directed behaviour in these mice was reduced rather than increased, suggesting that these mice were instead exhibiting some degree of real-time place avoidance.

We next examined whether contingent optogenetic self-stimulation was associated with alterations in the number of entries mice made into stimulation-paired zones (Fig. 2c, f). In eYFP control mice, exposure to light in the active zone did not impact how many entries mice made across the corner zones. In contrast, ChR2+ mice showed input-specific alterations in zone entry behaviour. Exposure to contingent stimulation of mPFC → NAcSh inputs altered the number of zone entries made (*F*_3,24_ = 3.7727, *p* = 0.0238, *ɳ*^2^ = 0.32) and increased active relative to inactive zone entries, indicating that these mice were obtaining rewarding stimulation by repeatedly exiting and re-entering the active stimulation zone. Alternatively, this zone entry effect was not found in vHPC → NAcSh mice (*F*_3,24_ = 0.5509, *p* = 0.6524) suggesting that these mice were preferentially obtaining rewarding stimulation by waiting in the zone for subsequent stimulations rather than leaving and re-entering. Exposure to BLA → NAcSh stimulation also had no effect on the number of entries made across the corner zones during acquisition testing (*F*_3,40_ = 0.7286, *p* = 0.5410). Interestingly, despite the observed reductions in time spent and stimulation received in the active zone in PVT → NAcSh mice, these mice failed to show significant reductions in the number of entries they made across the corner zones during the acquisition session (*F*_3,20_ = 0.9875, *p* = 0.4187).

### Input-specific differences in behaviour exhibited during reversal testing in ChR2 + mice

When the location of the active zone was changed during reversal testing (Fig. 3), eYFP control mice showed similar behaviour across the spatial arena as was found during the baseline and acquisition sessions, whereas input-specific behavioural differences were again detected in ChR2+ mice. While behaviour in mPFC → NAcSh mice was preferentially directed at the active zone during acquisition, this active zone-directed behaviour was not fully recapitulated during reversal as indicated by the lack of significant zone effects for the time in zone (Fig. 3d, *F*_3,24_ = 1.6413, *p* = 0.2062), stimulation time (Fig. 3e, *F*_3,24_ = 2.0868, *p* = 0.1286), and zone entry (Fig. 3f, *F*_3,24_ = 1.5569, *p* = 0.2257) metrics, raising the possibility that in addition to being rewarding, stimulation of this input may have impacted behaviour in these mice in a way that led to an inconsistent ability to maintain reward-directed behaviour. In contrast, vHPC → NAcSh mice showed full reversal of reward-directed behaviour indicated by increased time spent (Fig. 3d, vHPC: *F*_3,24_ = 16.0947, *p* < 0.0001, *ɳ*^2^ = 0.67) and stimulation received (Fig. 3e, vHPC: *F*_3,24_ = 21.1208, *p* < 0.0001, *ɳ*^2^ = 0.73) in the reversal zone, without altering entries made across the corner zones (Fig. 3f, *F*_3,24_ = 1.1030, *p* = 0.3672). Interestingly, the effects of BLA → NAcSh stimulation mice were more apparent in reversal than during acquisition testing, as these mice now exhibited significant differences in both time spent (Fig. 3d, *F*_3,40_ = 2.9637, *p* = 0.0435, *ɳ*^2^ = 0.18) and optogenetic stimulation received (Fig. 3e, *F*_3,40_ = 3.1728, *p* = 0.0344, *ɳ*^2^ = 0.33) across the corner zones. For PVT → NAcSh mice, time in zone (Fig. 3d, *F*_3,20_ = 5.2418, *p* = 0.0078, *ɳ*^2^ = 0.44), stimulation received (Fig. 3e, *F*_3,20_ = 3.2676, *p* = 0.0427, *ɳ*^2^ = 0.33), and zone entry metrics (Fig. 3f, *F*_3,20_ = 0.6659, *p* = 0.5828) were consistent with the acquisition session, and mice shifted their avoidance behaviour towards the new active zone.

### Pathway and contingency-dependent effects of stimulation on locomotor behaviour

**Fig. 4:**
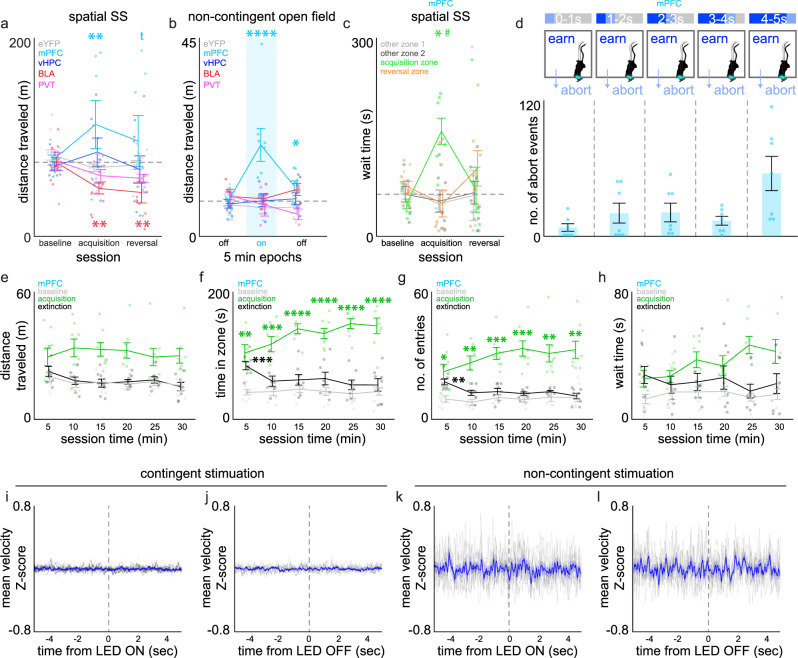
Disconnects between locomotor activity and reward-directed behaviour in mPFC → NAcSh mice. **a** Total distance traveled (m) during spatial self-stimulation (SS) sessions in eYFP (gray, *n* = 12 mice), mPFC → NAcSh (cyan, *n* = 7), vHPC → NAcSh (blue, *n* = 7), BLA → NAcSh (red, *n* = 11), and PVT → NAcSh (pink, *n* = 6) mice. Contingent stimulation (30 Hz) increased total distance traveled in mPFC→NAcSh and decreased it in BLA → NAcSh mice. **b** Total distance traveled (m) during 5 min epochs in eYFP (gray, *n* = 12), mPFC → NAcSh (cyan, *n* = 8), vHPC → NAcSh (blue, *n* = 6), BLA → NAcSh (red, *n* = 4), and PVT → NAcSh (pink, *n* = 3) mice. Non-contingent stimulation (30 Hz, 5 s on, 5 s off during on epoch highlighted in blue) increased distance traveled, but only in mPFC → NAcSh mice. **c** Total time spent waiting in the corner zones during the timeout period across contingent self-stimulation sessions in mPFC → NAcSh mice. **d** The number of active zone abort stimulation decisions made by mPFC → NAcSh mice during acquisition expressed across progressive 1 s time bins within the 5 s stimulation duration. **e**–**h** Behaviour exhibited in the across (5 min bins) baseline (gray), acquisition (green), and extinction sessions (black) in mPFC → NAcSh mice (*n* = 8). **e** Total distance traveled (m) across the entire arena, (**f**) Time spent in the stimulation-paired active zone, (**g**) Entries made into the stimulation-paired active zone, (**h**) Time spent waiting in active zones during the timeout period. **i**, **j** Velocity Z-scores during contingent stimulation onset (**i**) or offset (**j**) during acquisition in mPFC → NAcSh mice. **k**, **l** Velocity Z-scores during non-contingent stimulation onset (**k**) or offset (**l**) during acquisition in mPFC → NAcSh mice. Note that differences in velocity amplitudes between contingent and non-contingent conditions is likely related to differences in apparatus dimensions. Data is expressed as mean ± SEM. Analysis was done using mixed-model ANOVAs (**a**, **b**), two-way ANOVA (**c**), one-way ANOVA (**d**), two-way repeated measures ANOVAs (**e**–**h**), or paired two-tailed *t*-tests (**i**, **j**) tp < 0.1, *p < 0.05, **p < 0.01, ***p < 0.001, ****p < 0.0001 versus baseline session (contingent) or first off epoch (non-contingent). ^#^p < 0.05 versus other corner zones, within same session using Student’s (**a**, **b**) or Tukey’s (**c**, **f**, **g**) post hoc comparisons.

Although we found clear differences in reward-related behaviours described above, movement around the arena (Fig. 2b, Fig. 3b) and entry patterns (Fig. 2c, Fig. 3c) during the acquisition and reversal sessions did not appear consistent across the different ChR2+ inputs, so it is possible that alterations in general motor activity may underlie some of the behaviours being exhibited by mice in the spatial arena. To more directly examine this, we first assessed distance traveled during behaviour sessions across both input pathways and behaviour sessions using a mixed-model ANOVA with subject as a random factor. This analysis revealed a pathway x session interaction (Fig. 4a, *F*_8,76_ = 3.1330, *p* = 0.0041) indicating that activation of some, but not all pathways impacted motor behaviour during testing. Post-hoc comparisons within input pathways revealed that motor activity in mPFC → NAcSh was increased during self-stimulation sessions relative to the baseline session, whereas activity in BLA-NAcSh mice was decreased. In contrast, neither vHPC → NAcSh nor PVT → NAcSh exhibited alterations in locomotor activity during testing, suggesting that behaviours in these mice were not confounded by non-specific motor effects.

To further identify whether exposure to optogenetic stimulation produced non-specific effects on locomotor activity, we also assessed locomotor behaviour in a separate cohort of mice that received passive optogenetic stimulation in a contextually distinct spatial arena that was not contingent on any behaviour. Analysis here revealed a significant interaction between input pathway x stimulation period (Fig. 4b, *F*_8,56_ = 5.6410, *p* < 0.0001), suggesting again that stimulation of some, but not all pathways impacted motor behaviour. However, in this case only mPFC→NAcSh mice exhibited significant increases in motor activity during passive stimulation. Post-hoc comparisons indicated that mice increased their activity during the 5 min passive stimulation period and this effect that was reduced, but still apparent, after stimulation was discontinued. Together these data indicate a pathway-specific disconnect between locomotor activity exhibited in response to contingent versus non-contingent stimulation.

### Waiting in and leaving the stimulation-paired active zone in mPFC → NAcSh mice

As mPFC → NAcSh mice exhibited increased motor activity in conjunction with either contingent or non-contingent stimulation, it is possible that the repeated entries made into the active corner zones during the self-stimulation sessions were merely a consequence of non-specific changes in locomotor activity. For example, it is possible that activation of mPFC → NAcSh inputs transiently increased locomotor activity, causing the mice to leave the active zone during the stimulation bout, then returning to it after this effect wore off when stimulation was discontinued upon zone exit. If this were the case, we would expect mPFC → NAcSh mice to be unable to wait in the stimulation-paired zone past the 5 s. stimulation bout, and instead would leave the zone (i.e., have an abort event) at a consistent duration after the onset of stimulation. However, analysis of waiting and leaving behaviour indicated this was not the case. A two-way ANOVA analysis of wait time (i.e., time spent in the zones during the timeout period) across sessions in these mice revealed a significant session x zone interaction on wait times (Fig. 4c, *F*_6,72_ = 3.1649, *p* = 0.0082, *ɳ*^2^ = 0.25). Post-hoc comparisons further revealed that these elevations in wait time occurred selectively within the active zone during the acquisition session, despite locomotor activity being elevated at this time. Thus, mice were indeed capable of remaining in the active zone beyond the initial 5 s of stimulation. A one-way ANOVA analysis of the number of abort events across 1 s time bins during the 5 s stimulation bouts also indicated that these events occurred across varying times points after the initiation of stimulation (Fig. 4d, *F*_4,30_ = 4.3049, *p* = 0.0072, *ɳ*^2^ = 0.36). Taken together, these data indicate that activation of mPFC → NAcSh mice inputs was producing purposeful changes in locomotor activity that were associated with the repeated actions being taken in these mice across the self-stimulation sessions.

### Preservation of behaviour in mPFC → NAcSh mice when stimulation is discontinued

To further confirm that locomotor activity was associated with repeated actions in mPFC → NAcSh mice, we also examined acquisition and extinction of behavioural strategies over time in a separate cohort of mice (*n* = 8). Two-way repeated measures ANOVAs were used to analyze overall locomotor behaviour and active zone-specific behaviour over time during these sessions (Fig. 4e–h). We found a significant effect of session on locomotor activity (Fig. 4e, *F*_2,21_ = 11.07, *p* = 0.0005, *ɳ*^2^ = 0.88), with locomotor activity being elevated during the acquisition compared to baseline or extinction sessions. We also found significant session *x* time interaction effects for time spent in the active zone (Fig. 4f, F_10,105_ = 3.887, *p* = 0.0002, *ɳ*^2^ = 0.37) and active zone entries (Fig. 4g, *F*_10,105_ = 2.722, *p* = 0.0052, *ɳ*^2^ = 0.26), with a trend for interaction on active zone wait times (Fig. 4h, *F*_10,105_ = 1.877, *p* = 0.0565). In contrast to the enhanced locomotor activity, these measures developed over the course of the session, and post hoc comparisons indicated that elevation in the time in zone and zone entry metrics were still expressed early during extinction testing, then extinguished over time. Finally, we used SLEAP^35^ pose and position analysis to identify any velocity changes that were directly associated with stimulation onset and/or offset during active (Fig. 4i, j, Supplementary Fig. 5) or passive stimulation (Fig. 4k, l, Supplementary Fig. 5) and found no evidence for any consistent alterations in velocity that were temporally paired with either stimulation onset or stimulation offset. Taken together, these data suggest that activation of mPFC → NAcSh inputs were reinforcing purposeful actions made by the mice rather than simply producing non-specific elevations in locomotor activity.

### BLA → NAcSh mice are sensitive to time investments made in the active zone

Given discrepancies between the expression of place preferences and locomotor effects during testing between vHPC → NAcSh mice and BLA → NAcSh mice, we also sought to better distinguish behaviours across these two inputs by examining another behavioural metric afforded by this foraging paradigm: relationships with the passage of time. Because both vHPC → NAcSh mice and BLA → NAcSh appeared willing to remain in the active zone during the timeout period to receive subsequent stimulations, we assessed the relative probability these mice would stay in the active zone during the timeout period as a function of time already waited (p(stay), Supplementary Fig. 6a,b). While vHPC → NAcSh mice only showed elevations in this metric during reversal testing relative to the baseline session, this time-dependent tendency to remain in the active zone specifically during the timeout period was consistently increased during both acquisition and reversal in BLA → NAcSh mice. Further, this metric was significantly elevated in BLA → NAcSh compared to vHPC → NAcSh during both acquisition and reversal sessions (Supplementary Fig. 6c). This relative increase in sensitivity to time investments (i.e., ‘sunk costs’) was also seen in detrended curves and resulting slopes when data was pooled across inactive (baseline) and active (acquisition and reversal) sessions (Supplementary Fig. 6d, e). Thus, unlike the vHPC → NAcSh mice who appeared to develop a more straightforward place preference, the decision of BLA → NAcSh mice to remain in the reward-associated context in the absence of any ongoing stimulation was associated with a relatively stronger resistance to leave with the passage of time.

### Quadruple dissociation of behaviours across NAcSh inputs

**Fig. 5:**
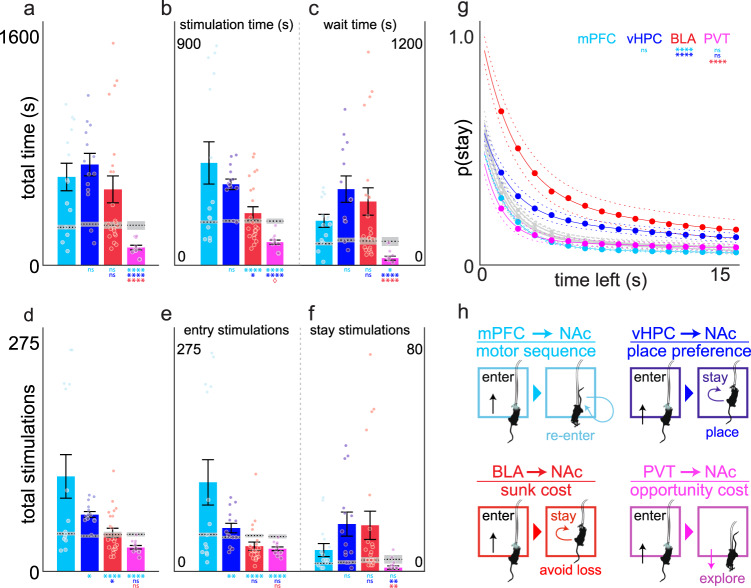
Direct comparison of optogenetically-induced behavioural strategies across NAcSh inputs. Different NAcSh input groups are depicted by different colours throughout: mPFC → NAcSh (cyan, *n* = 7 mice), vHPC → NAcSh (blue, *n* = 7 mice), and BLA → NAcSh (red, *n* = 11 mice), and PVT → NAcSh (pink, *n* = 6 mice). **a** Total time spent in active stimulation zones during testing. mPFC→NAcSh, vHPC→NAcSh, and BLA → NAcSh mice spent similar amounts of time in active zone, while PVT → NAcSh mice spent relatively less time in the active zones. **b**, **c** Time spent being actively stimulated (stimulation time, **b**) versus waiting during the timeout period (wait time, **c**). The relative amount of time spent being stimulated versus waiting differed across inputs. **d** The total number of stimulation bouts was significantly higher in mPFC → NAcSh mice compared to other groups, which reflects the high number of stimulations by direct zone entry (**e**). In contrast, vHPC→NAcSh and BLA → NAcSh mice earned higher numbers of stay stimulations by prolonged lingering during the timeout period (**f**). **g** BLA → NAcSh mice were more likely to remain in the active zone throughout the timeout period, distinguishing it from other groups. **h** Proposed credit assignment functions for each set of NAcSh excitatory inputs. Figures represent pooled data (mean ± SEM) from active corner zones during acquisition and reversal sessions. Mixed-model ANOVAs were used to identify pathway x zone (pooled active or inactive) interactions and Tukey’s post hoc tests (outlined in Supplementary Fig. 8) were used for between-pathway comparisons within pooled active zones. Inactive zone data are depicted in Supplementary Fig. 9. Dashed lines represent pooled data from the baseline session and are presented for comparison but were not included in the analysis. *p < 0.05, **p < 0.01, ***p < 0.001, ****p < 0.0001 versus other pathways as specified.

Finally, to compare behaviour more directly across NAcSh inputs, we pooled behaviour metrics in the active zones across both acquisition and reversal tests and compared them against pooled metrics in the inactive zones during these tests (Fig. 5, Supplementary Fig. 8; baseline values are depicted for comparison but were not included in the analysis). Mixed-model ANOVA analysis revealed a significant zone (active or inactive) x brain pathway interaction for time in zone (Fig. 5a, *F*_3,213_ = 13.4753, *p* < 0.0001), stimulation time (Fig. 5b, *F*_3,213_ = 19.2294, *p* < 0.0001), wait time (Fig. 5c, *F*_3,213_ = 10.8662, *p* < 0.0001), total stimulations (Fig. 5d, *F*_3,213_ = 16.1441, *p* < 0.0001), entry stimulations (Fig. 5e, *F*_3,213_ = 18.1867, *p* < 0.0001), stay stimulations (Fig. 5f, *F*_3,213_ = 5.3269, *p* = 0.0015), and p(stay) metrics (Fig. 5g, *F*_3,224_ = 31.0202, *p* < 0.0001) indicating that across-pathway differences occurred primarily in the active zones. Post-hoc comparisons of behaviour in the active stimulation zones across pathways for these metrics (Supplementary Fig. 7) clearly showed PVT → NAcSh mice spent less time in the stimulation-paired zones compared to mPFC → NAcSh, vHPC→NAcSh, or BLA → NAcSh mice (Fig. 5a). Despite spending similar amounts of time in the active zone, we found input-specific differences in the amount of optogenetic stimulation received (Fig. 5b) and timeout-related wait times (Fig. 5c) across mPFC → NAcSh, vHPC → NAcSh, and BLA → NAcSh inputs. While mPFC → NAcSh mice tended to self-administer optogenetic stimulation by repeatedly entering the active zone (Fig. 5e), vHPC → NAcSh and BLA → NAcSh mice were more likely to wait in the active stimulation zone to obtain additional stimulation (Fig. 5c, f). Only BLA → NAcSh mice showed increased sensitivity to the passage of time spent waiting in the active zone during timeout periods relative to the other inputs (Fig. 5g). Notably, the input-specific differences in behaviour we observed were still apparent, albeit weaker, when assessed in separate groups of mice at lower frequencies (Supplementary Fig. 8), strongly suggesting that source, rather than amount, of excitatory input to the NAcSh mediated the effects we observed in this study.

## Discussion

In the present study we found that optogenetic stimulation of individual excitatory inputs to the NAcSh results in the expression of distinct behavioural outputs in a contextually based spatial self-stimulation task that extends beyond the simple rewarding/aversive dichotomy. We further provide evidence that input-specific changes in locomotor activity during self-stimulation sessions reflect more purposeful alterations in activity rather than non-specific response to the optogenetic stimulation, consistent with previously demonstrated disconnects across NAcSh inputs in stimulation-related locomotor activity and depression-like behaviour^36^. This quadruple dissociation of reward-related strategies highlights how integration of multiple excitatory inputs to the NAcSh guides motivated behaviour and response selection.

The source and target nuclei investigated in the present study comprise an integrative network that constructs outcome predictions and guides the selection of goal-directed behaviour^16,37^. Within this network, the ventral striatum serves as a key site of convergent input from cortical and limbic regions that is critical for integration of information and regulation of behavioural outputs^16,38^. However, the question remains what specific information may be relayed by the individual inputs to drive the behaviours we observed. One intriguing possibility is that individual NAcSh inputs could assist in such information integration by providing different valuation information to the NAcSh related to specific credit assignment functions. Credit assignment is a retrospective process that influences future predictions about outcomes that then guides decision-making and response selection. These functions play a critical role in reward valuation, reinforcement learning, computational decision-making processes, and goal-directed behaviour^39^. In foraging animals, these functions can contribute to ongoing cost-benefit analyses and help hone behaviour towards a survival-favorable outcome by reinforcing behaviours that increase an animal’s ability to obtain rewards and/or avoid danger. Furthermore, both incentive salience^40^ and Pavlovian action^41,42^ reinforcement learning paradigms involve the credit assignment problem^43^—given a recent positive or negative experience, how should one weigh sets of convergent stimuli when assigning credit?

The ventromedial mPFC plays an important role in executive control and is involved in reward representation, value-based decision making, action selection, response inhibition, attention, task switching, and habit formation^16,44–47^. The infralimbic cortical area we targeted in the present study, which preferentially innervates the NAcSh^16^, has been further implicated in response-outcome encoding^47,48^ and synaptic plasticity at infralimbic inputs to the NAcSh selectively impacts re-evaluative, but not deliberative, aspects of decision-making processes^9^. While both the mPFC and ventral striatum have previously been identified as being involved in credit assignment processes^49–51^, to our knowledge this is the first time that direct connections between the mPFC and NAcSh have specifically been implicated. In our case, the behaviour exhibited by mPFC → NAcSh mice is most consistent with this input assigning value to recent actions. These mice made repeated entries into the stimulation-paired zone during acquisition but also showed more variable active zone entry behaviours during reversal testing, consistent with work indicating that the mPFC, and particularly the infralimbic area targeted here, is important for strategy shifting but is less involved in reversal learning^52^. If mPFC → NAcSh stimulation promoted recent action sequences, we would predict that mice would exhibit repeated active-zone entry during acquisition testing as entering the stimulation-paired zone was the most recent action exhibited prior to stimulation being delivered. This repeated entry into the original acquisition zone would presumably persist initially during reversal, but when this action was not reinforced by further stimulation, mice would likely take different subsequent exploratory search approaches in the arena, and these varying motor sequences would then be reinforced when mice entered the new stimulation-paired reversal zone. Indeed, when mice were given an extinction instead of a reversal session, they did initially preserve their entry behaviour towards the previously active zone, but this behaviour quickly dissipated in the absence of further stimulation. This latter finding is consistent with the role of the infralimbic cortex in learning new stimulus-reward associations during extinction training^53^. The reinforcement of action-based credit assignment functions through the NAcSh may also provide a way that the infralimbic cortex can promote goal-directed response vigor^54^ while also suppressing unwanted actions^55^.

In addition to assigning credit to beneficial actions, animals must be able to link specific actions and outcomes with associated environmental stimuli. The hippocampus is a critical for encoding such contextual information about the environment^56^ and the vHPC subregion is known to be important for spatial navigation, context-based associative learning, and emotional and affective processing^57^. The vHPC has also been implicated in value-based decision making^58^ as well as credit assignment processes^59^, although which outputs of the vHPC are critical and what specific valuation information is provided is less clear. The spatial arena we utilized in the present study was contextually rich in that each corner had distinct borders and contextual markers, so these features were the primary ones the animals had to use to identify the location of the stimulation-paired corner. Compared to the other inputs, vHPC → NAcSh stimulation produced the most consistent and selective place preferences for these stimulation-paired corners during both acquisition and reversal testing. These mice also lingered in the stimulation-paired corners during the timeout period in both acquisition and reversal sessions and were more likely to obtain additional bouts of stimulation compared to mPFC → NAcSh mice. Together, these findings indicate that instead of assigning credit to recent actions, vHPC → NAcSh mice were assigning value to the stimulation-paired context itself. Such context-based credit assignments would be consistent with the proposed role of vHPC inputs to the nucleus accumbens in model-based spatial navigation, goal-directed behaviour, and spatial reversal learning^33,60–64^ and would additionally provide a way for vHPC inputs to influence intertemporal choice, cost-benefit decision-making, and approach-avoidance behaviour^58,65,66^.

The BLA is important for incentive and motivational processing, associative learning, behavioural flexibility, outcome-specific representations, intertemporal choice, and cost-benefit decision-making processes^31,67^. While BLA projections to the NAc core subregion are important for outcome devaluation, projections to the NAcSh are more involved in outcome-specific Pavlovian-to-instrumental transfer and associative learning that pairs environmental cues and reward-related outcomes^67–69^. Notably, in our task we did not pair temporally discrete cues with optogenetic stimulation, so this may be one reason why BLA → NAcSh mice self-administered less optogenetic stimulation and were relatively slower to show reward-directed behaviour compared to mPFC → NAcSh and vHPC → NAcSh inputs. However, it is more likely that these effects were primarily driven by the increased sensitivity to time investments that we identified in BLA → NAcSh mice. The assignment of credit to time investments (sunk costs) would also more readily explain why BLA → NAcSh mice showed reductions in locomotor activity, but only when stimulation was contingent on the animal’s behaviour. While there is limited evidence for BLA involvement in credit assignment processes^70^, such credit assignments would be very consistent with an alteration in intertemporal choice and an aversion to leaving a reward-associated environment that has been described in foraging animals and humans^71–78^. Such credit assignments could also explain how BLA → NAcSh stimulation could impact both spatial and temporal aspects of cost-benefit decision-making processes^79–81^.

Interestingly, only PVT → NAcSh mice showed reductions rather than elevations in active zone- directed behaviour during self-stimulation sessions. However, they did not avoid entering the zone entirely, suggesting that PVT → NAcSh stimulation produced a more complex interaction with the behavioural task that prevented the expression of full avoidance behaviour. Given the heterogeneous nature of the PVT and its implications in arousal and both appetitive and aversive behaviour^22,23,82^, this complexity may reflect heterogeneity of these projections at either the pre- or post-synaptic level. For example, activity in anterior PVT projections is more associated with appetitive behaviour, whereas projections from posterior PVT are more often associated with aversive behaviour^23^. While we chose to target coordinates that have previously been shown to have dense projections to the NAcSh, produce real-time place aversion upon optogenetic stimulation, and undergo plasticity after exposure to addictive drugs^24^, it has been also been demonstrated that optogenetic activation of more anterior PVT regions promotes appetitive behaviour^21,83^. Given the spread of our viral injections across both anterior and posterior regions of the PVT (Supplementary Fig. 1), it is possible we are accessing both types of pathways. On the post-synaptic side, because PVT → NAcSh projections synapse onto both dopamine receptor type 1 (D1+) and type 2 (D2+) containing MSNs^25^, contingent stimulation of PVT → NAcSh inputs may affect both post-synaptic cell types, resulting in both appetitive and aversive-like responding. However, as all PVT mice showed some level of avoidance of the stimulation-paired zone, these possibilities cannot fully explain the behaviour exhibited by these mice. Alternatively, another credit assignment function, opportunity cost, could explain the behaviour we observed in PVT → NAcSh mice. Activation of this credit assignment function would decrease the value of remaining in the stimulation-paired corner and increase the value of exploring other options. The potential opportunity costs to increase exploratory behaviour seems particularly likely given the low risk and low opportunity cost of doing so in our spatial task^84,85^. However, after exploring the other corner zones and finding no reward opportunities there, mice would presumably return to the stimulation-paired zone to reassess the situation, which is consistent with our observations (Fig. 2c, f; Fig. 3c, f).

Given the present data, we propose a potential pathway mechanism for how different excitatory inputs provide convergent valuation information to the NAcSh to guide behavioural strategies during reinforcement learning (Fig. 5h). We propose that each excitatory input to the NAcSh reinforces different credit assignment functions, with mPFC → NAcSh assigning value to recent actions; vHPC → NAcSh inputs assigning value to spatial contexts, BLA → NAcSh inputs assigning value to time invested (sunk costs), and PVT → NAcSh inputs assigning value to opportunities. While credit assignment processes in reinforcement learning are typically discussed in relation to striatal dopamine function^86^, our data strongly suggest that striatal glutamate release at individual NAcSh inputs is also critically involved. While specific local pathway effects and network-wide dynamics remain to be elucidated, the convergence of such information in the NAcSh has important implications for both reinforcement learning and value-based decision-making processes and provides mechanistic insight into how behaviours could be selected and adapted during foraging. Given that different experiences will access these different inputs, and that these pathways could be separately affected by diseases or trauma, our findings provide insight regarding how plasticity at different NAcSh inputs contributes to both adaptive and maladaptive learning found across multiple psychiatric disorders.

## Methods

### Subjects

A total of 125 adult male C57Bl/6 J mice (Jackson Laboratories) were used. Mice were ~6 weeks of age (18–22 g) at the beginning of study and were housed in a temperature and humidity-controlled vivarium under a 12-h light-dark cycle (lights on at 0600). All mice were habituated to the vivarium for at least 5 days before undergoing any surgical procedures. Mice were group housed until fiber implant surgery at which time they were single housed. This approach was done to minimize stress due to single housing as well as to preserve the integrity of the implants. All surgical and experimental procedures were approved by the University of Minnesota Institutional Animal Care and Use Committee and followed guidelines of the American Association for the Accreditation of Laboratory Animal Care.

### Experimental design

The overall experimental design is illustrated in Fig. 1. Mice underwent one surgery to inject a viral vector that expresses the blue-light activated Channelrhodopsin (ChR2) under a CamKIIα promoter to drive expression in glutamatergic projection neurons terminating in the NAcSh, followed by a second surgery to implant an optical fiber above ChR2+ terminal regions in the NAcSh. After recovery, mice underwent three consecutive days of behavioural testing to examine pathway-specific self-stimulation behaviour.

### Surgical procedures

Mice were anesthetized with a ketamine/xylazine cocktail (100 and 10 mg/kg, IP) and were given bilateral injections of AAV2-CamKIIa-eYFP (control virus) or AAV2-CamKIIa-ChR2(H134R)-eYFP (University of North Carolina Vector Core) using a 5 µl Hamilton syringe with a 29-gauge needle. Injections were targeted to the infralimbic cortex (mPFC: 0°, A/P + 1.8, M/L + /− 0.4, D/V −3.1 from Bregma), ventral hippocampus (vHPC: 0°, A/P −3.08, M/L + /−2.9, D/V −4.25 from Bregma), basolateral amygdala (BLA: 0°, A/P −1.3, M/L + /−3.1, D/V −4.9 from Bregma), or paraventricular nucleus of the thalamus (PVT: 4°, A/P −1.2, M/L + /−0.1, D/V −3.2 from Bregma). Viral volume was 0.5 µl/side, expect for PVT injections which were 0.25 µl/side. PVT injections were given bilaterally to mimic bilateral injection parameters of the other brain regions and but were angled to avoid hitting the midline ventricular areas. Coordinates were identified using from a mouse brain atlas^87^ and were consistent with previous research that targeted these regions for assessment of real-time place preference or instrumental self-stimulation procedures^24,26,27,88^. Approximately 3–4 weeks after virus injection, a second surgery was used to place custom-made optical fiber implants (200 µm core, 0.66 NA fiber; 230 µm ID ferrules, ThorLabs). Fibers were implanted bilaterally directly above the nucleus accumbens shell (NAcSh: 14˚, A/P + 1.5, M/L + /−1.63, D/V −4.1 from Bregma/skull). Fibers were secured to the skull using stainless steel machine screw anchors (0.0625'') and Geristore dental acrylic. When not in use, fibers were kept capped (Precision Fiber Products) to prevent damage. Mice were given 5–10 days of recovery before behavioural testing. We chose to target the NAcSh subregion given that it is involved in reward valuation, is a key region where cortical and limbic inputs converge^11,17,89,90^, and that plasticity in this region has been implicated in multiple psychiatric disorders^12,14,15,38^^.^ Optogenetic stimulation of glutamatergic mPFC^27^, vHPC^27^, and BLA^26,27,88^ inputs to the NAcSh subregion have also been demonstrated to induce real-time place preferences and reinforce instrumental behaviour, whereas excitatory PVT inputs to the NAcSh cause real-time place avoidance^24^, but also can promote instrumental responding^25^. Furthermore, as the NAcSh is a site thought to be critical for integration of information^38^, we wanted to further explore whether different sources of glutamate input to this subregion matter for reward-directed behaviour^28^.

### Optogenetic self-stimulation task

To examine optogenetic self-stimulation behaviour, we developed an open-field arena (20''x 20'') with isolated corners zones containing different contextual cues (e.g., triangles, dots, horizontal or vertical lines). This approach allowed mice to discriminate between the different corner zones and provided space in the arena where mice could explore that was free of contextual marker and/or stimulation consequences. Mice underwent three consecutive days of testing as described below.

#### Baseline

Mice were initially habituated to the spatial environment during a 30 min baseline session. In this session, mice were allowed to freely explore the spatial arena, but entry into any of the corner zones was without consequence. The amount of time spent in each zone during this baseline was measured. Neither the most preferred nor the least preferred zone was chosen as the active acquisition or reversal zone for subsequent self-stimulation tests to avoid artificial increases or decreases in time spent in the zone that were unrelated to the optogenetic stimulation itself. Thus, the 2nd or 3rd preferred zones were chosen as active zones and their designation as “acquisition” or “reversal” zones was counterbalanced across experimental groups. Zone assignments for the two other corner zones were also counterbalanced prior to acquisition testing.

#### Acquisition

Separate groups of mice were assessed for self-stimulation behaviour at a particular frequency (30 Hz, *n* = 64, 20 Hz, *n* = 30, or 10 Hz, *n* = 31) during a 30 min acquisition test. PlexBright LEDs (Plexon) were used to deliver blue light (465 nm, 5 ms pulse width, 10–15 mW, ~4–6 mW/mm^2^ at target tissue) bilaterally through patch cables (200 µm, 0.66 NA, Plexon) that were connected to brain implants using light shielded zirconia sleeves (1.25 mm OD, Plexon). A single corner zone was designated as the active stimulation zone and entry into this zone triggered an active LED, whereas entry into the other 3 “non-active” zones triggered an inactive LED (mock stimulation). Optogenetic stimulation in the active zone ended immediately upon zone exit, or after a maximum of 5 s if a mouse remained in the active zone. A 15 s timeout period was initiated after 5 s of stimulation after which another train of stimulation was initiated. Thus, mice could bypass the 15 s timeout period by exiting and re-entering the active zone or wait the duration of the 15 s timeout periods to receive additional stimulation. This approach allowed mice to titrate stimulation levels as well as provided mice with different strategy options for stimulation. It also allowed us to collect additional behavioural data beyond the total time spent in each corner zone including the actual amount of optogenetic stimulation received (real or mocked stimulation time), the number of zone entries that were made, the time spent lingering in a zone during the timeout, and the number of times mice received more than one stimulation train per entry (stay stimulations).

#### Reversal

Mice received a 30 min reversal test to further examine the expression of the behavioural strategy observed during acquisition testing. The test had the same parameters as during acquisition, except that the previously active zone (acquisition zone) was deactivated, and a different zone was activated (reversal zone).

#### Extinction

To identify whether behaviours exhibited during acquisition maintained after discontinuation of contingent stimulation in the spatial arena, a separate cohort of mPFC → NAcSh (*n* = 8) mice underwent baseline and acquisition sessions as described above, but instead of reversal testing they were given a single 30 min extinction session where stimulation availability was discontinued.

### Non-contingent passive stimulation

Mice were assessed for locomotor activity in response to passive (30 Hz, 5 ms pulse width) optogenetic stimulation in a distinct open-field apparatus which consisted of a beige rectangular (22 × 42 x 20 cm) box with corn cob type bedding on the bottom and no contextual cues available. eYFP (*n* = 12), mPFC → NAcSh (*n* = 8), vHPC → NAcSh (*n* = 6), BLA → NAcSh (*n* = 4), and PVT → NAcSh (*n* = 3). Mice were initially habituated to the apparatus for 30 min. They then underwent three stimulation periods (5 min each, off-on-off), with optogenetic stimulation being delivered during the middle period (5 s on, 5 s off). Stimulation parameters were set to provide more continual stimulation than was received in the spatial active self-stimulation task, but still mimic stimulation bout timing parameters set in this task. This spacing also allowed us to directly assess locomotor velocity changes that were temporally paired with either onset or offset of passive stimulation.

### Histology

After behavioural testing, mice were deeply anesthetized with pentobarbital (Fatal Plus, 390 mg/ml) and transcardially perfused with 1x phosphate buffered saline (PBS) followed by 4% paraformaldehyde. Brains were removed, placed in vials containing 4% paraformaldehyde overnight at 4 °C, and were then transferred to a 20% (1 day) and then 30% sucrose solution until brains had sunk and ready for slicing. A sliding microtome (Leica Biosystems) was used to cut 30 µm coronal sections, which were subsequently slide mounted and imaged with an epifluorescent microscope (Leica) to identify location of ChR2 virus and optical fiber placement. Only data from mice with correct virus and fiber placement were used for analysis.

### Statistics and reproducibility

Behavioural data was collected with ANY-maze (Stoelting Co.) and processed with ANY-maze, Matlab, or Python. JmpPro13/15 or GraphPad Prism9 was used for graphing and data analysis. Dependent variables included time spent in corner zones, time receiving actual or mock stimulation (active vs inactive zones, stimulation time), number of zone entries made (number of entry stimulations), wait time (time spent in corner zone when not being stimulated), number of stay stimulations (times mice received more than one stimulation per entry into a zone, i.e., stayed in zone through entire timeout period), number of abort events, distance traveled (m), velocity (z-scores), and probability of remaining in the zone as a function of time invested (p(stay)). One-way analysis of variance (ANOVA) with corner zone as a fixed factor was used to assess within-pathway behaviour across the four corner zones within each testing session (baseline, acquisition, reversal). A one-way ANOVA with time bin as a fixed factor was used to assess abort events in the acquisition zone during acquisition in mPFC → NAcSh mice. Two-way ANOVAs with zone and session as fixed factors were utilized for within-pathway comparisons of wait time behaviour. Two-way repeated measures ANOVAs with session and time as fixed factors and time as the repeated measure were used to assess behaviour over time and across sessions for extinction experiments. Mixed-model ANOVAs with pathway and session/epoch as fixed factors and subject as a random factor were used for across-pathway and session/epoch comparisons of locomotor behaviour. Paired *t*-tests were used to analyze z-score velocity data averaged 5 s before and after stimulation onset and/or offset. Mixed-model ANOVAs with pathway, session, and time left as fixed factors and subjects as a random factor were used for across-pathway and session comparisons of p(stay) values in vHPC and BLA mice. Mixed-model ANOVAs with pathway and zone type as fixed factors and subject as a random factor were used also for across-pathway comparisons of pooled behavioural metrics across zones (active or inactive) during active stimulation sessions (i.e., acquisition and reversal, baseline data presented for comparison, but not included in these analyses). Significant main or interaction effects were followed by either Student’s *t*-tests (for a priori comparisons) or Tukey’s post hoc tests. Significance level for main and interaction effects was set at *p* < 0.05. Effect sizes were calculated as follows: *ɳ*^2^ = SS_effect_/SS_total_.

### Reporting summary

Further information on research design is available in the Nature Portfolio Reporting Summary linked to this article.

## Supplementary information

Supplementary Information

Description of Additional Supplementary Files

Supplementary Data 1

Reporting Summary

## Data Availability

Source data for the main and supplementary figures is provided as Supplementary Data 1. All related raw data and processing codes are available upon reasonable request.
