# Supplementary material for: A quadruple dissociation of reward-related behaviour in mice across excitatory inputs to the nucleus accumbens shell
Source: Commun Biol. 2023 Jan 30;6:119. doi: 10.1038/s42003-023-04429-6 (PMC9886947; doi:10.1038/s42003-023-04429-6)
Supplement: Supplementary file 1 — Supplementary Information [file 42003_2023_4429_MOESM1_ESM.pdf]

SUPPLEMENTARY FIGURES

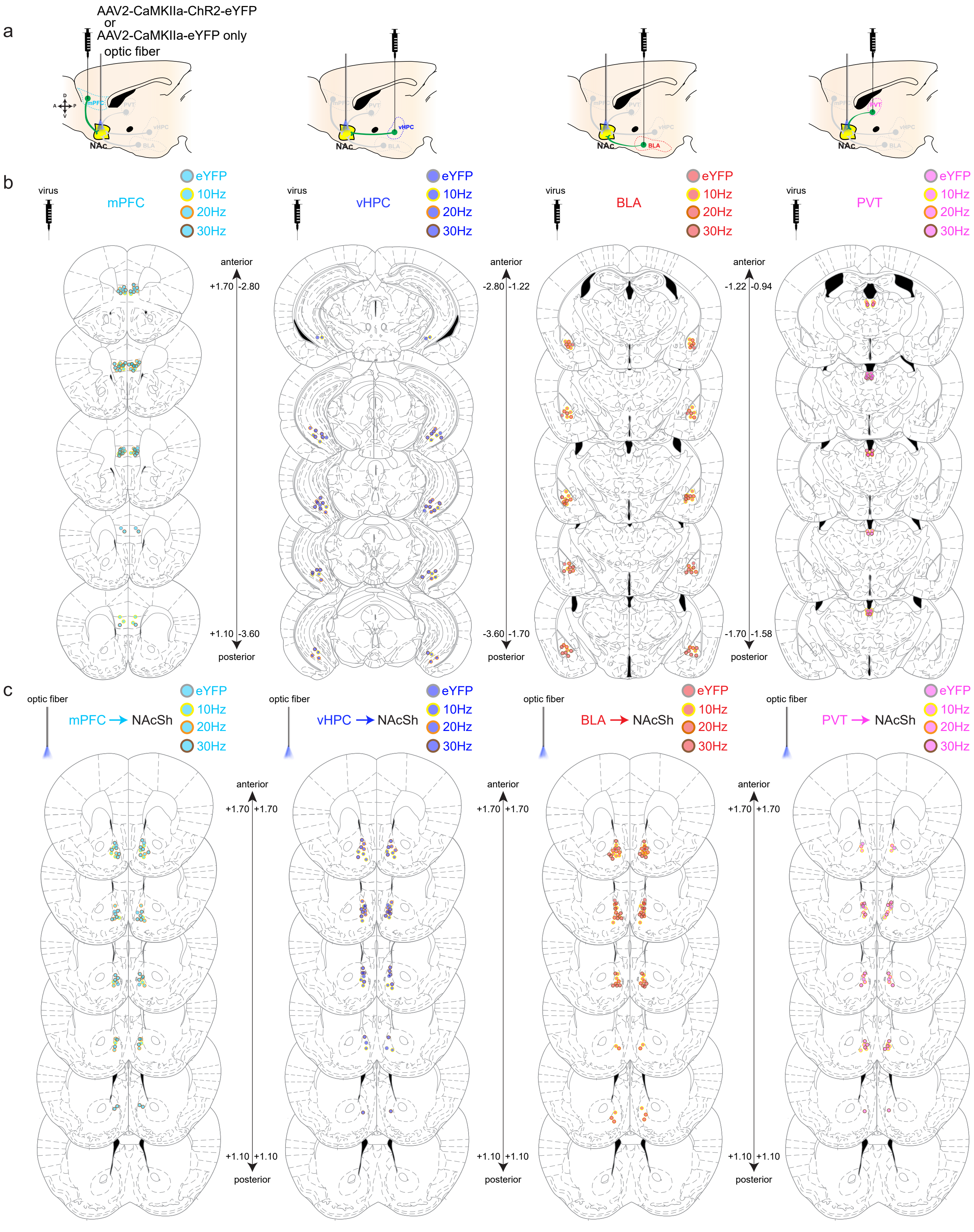

Supplementary Figure 1. Viral vector and optical fiber targeting in experimental mice. (a) Targeting approach for mPFC→NAcSh, vHPC→ NAcSh, BLA→ NAcSh, and PVT→ NAcSh experimental groups. **(b)** Coronal sections showing location of viral transfection injection site in experimental animals used in the main text (30 Hz) and in Supplementary Fig. 8 (20 and 10 Hz). **(c)** Coronal sections showing location of fiber tips in the NAcSh in experimental mice shown in (b). Anterior-posterior gradients are shown with colour coding indicating both pathway targeted and frequency used for behavioural testing.

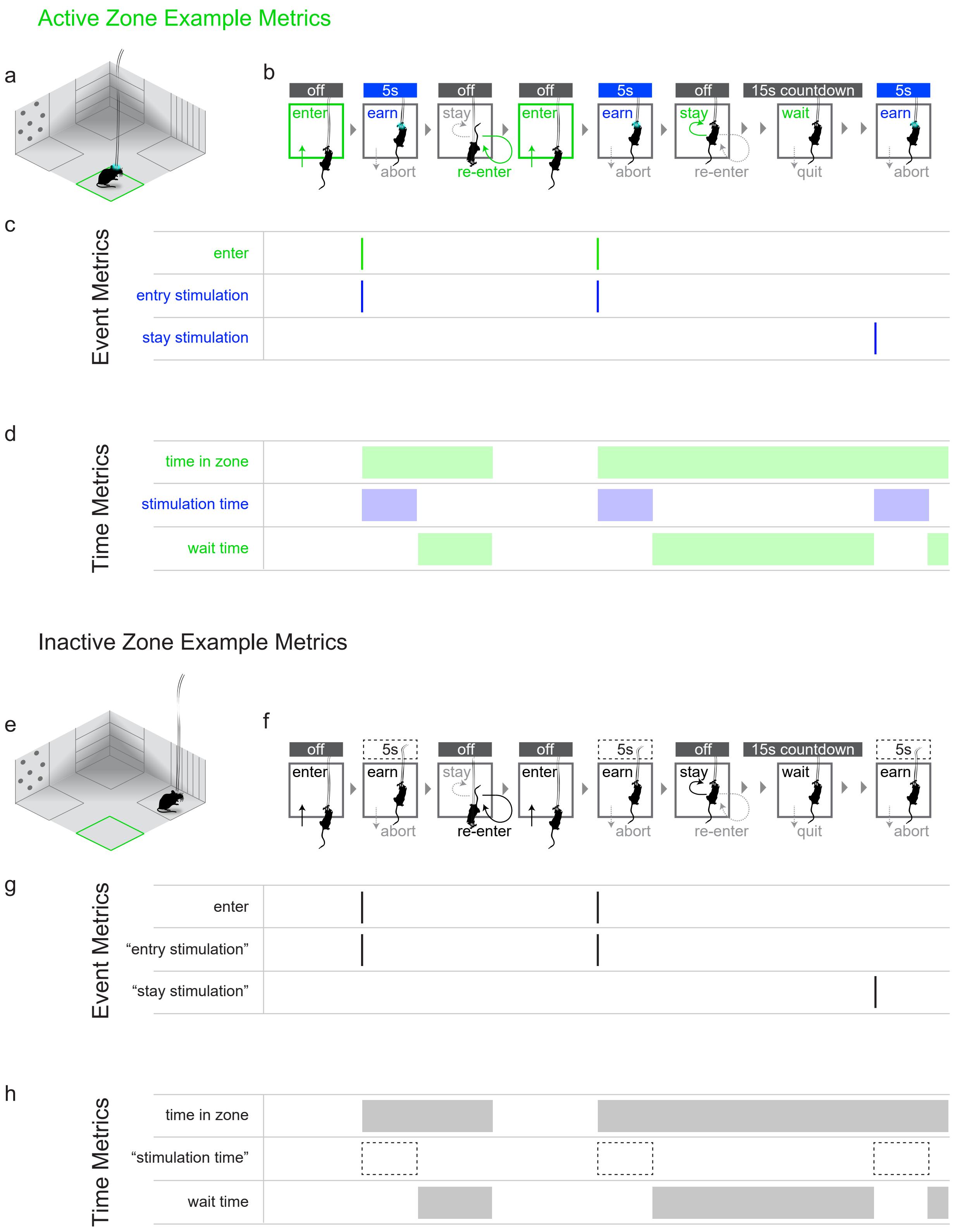

Supplementary Figure 2. Illustration of event and time metrics. (a) Schematic of the testing arena depicting a mouse receiving optogenetic stimulation in the active zone (green outline). **(b)** Example sequence of behavioural events, showing a total of two active zone entries and three optogenetic stimulations, two of which are triggered by entries, one of which is triggered following a 15 sec stay period. None of the optogenetic stimulations in this example (5 sec each) are terminated early by an abort decision. **(c)** Depiction of the event metrics (entries, entry stimulations, or stay stimulations) in (b) over time used to calculate data shown in Fig. 2e-f and Fig. 3c-d. **(d)** Depiction of the time metrics (time in zone, stimulation time, or wait time) in (b) over time used to calculate data shown in Figure 2d and Figure 3a-b. **(e-h)** Same as above except for behaviour measured in regard to inactive zones. Because mice do not actually receive stimulation in the inactive zone, (e-h) depict how equivalent metrics are measured for comparison purposes.

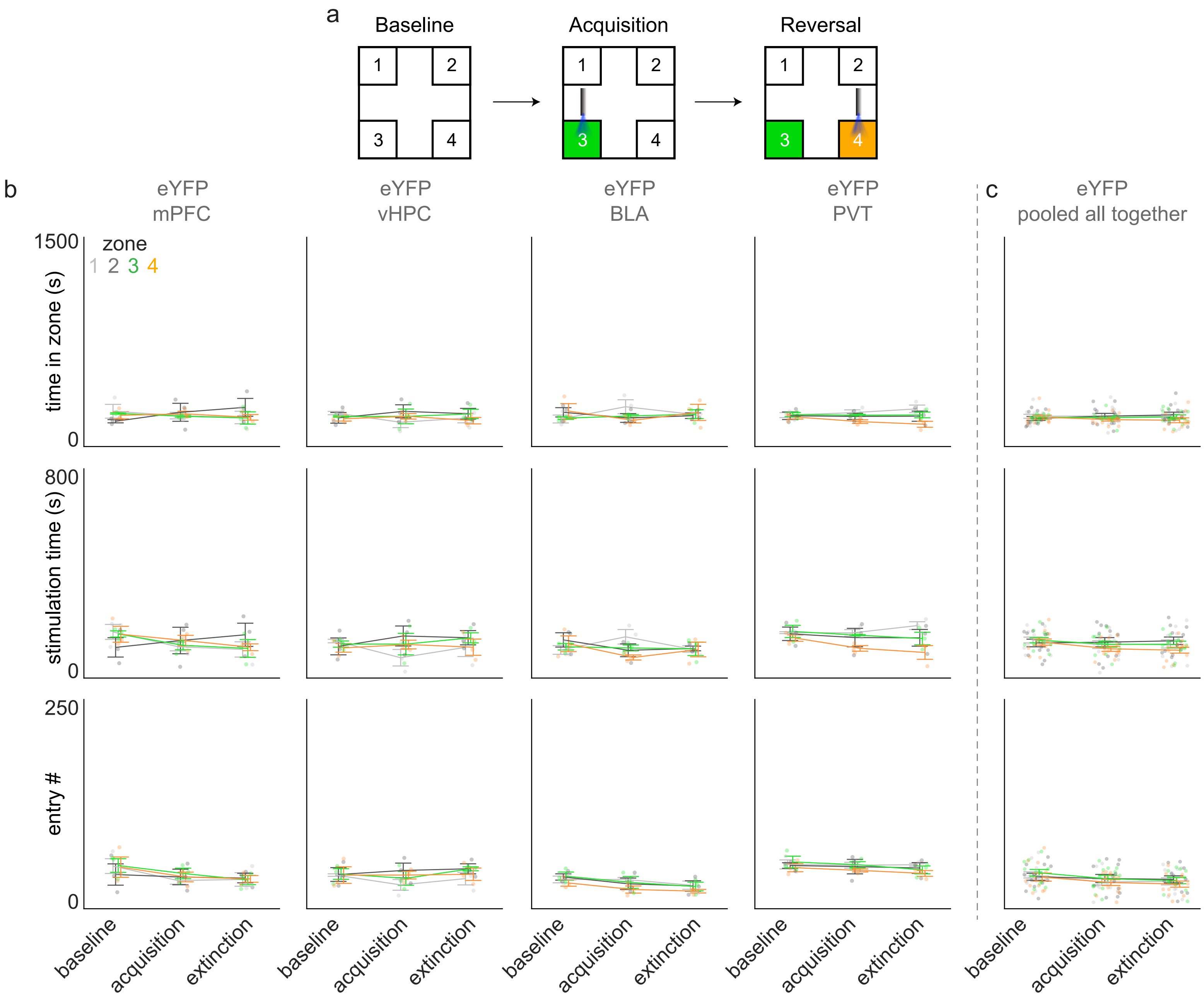

Supplementary Figure 3. No differences in behaviour across pathway or session in eYFP mice. (a) Spatial self-stimulation testing paradigm. **(b)** Mice injected with eYFP in the mPFC (n = 3), vHPC (n = 3), BLA (n = 3), or PVT (n = 3) and optical fibers in the NAcSh (all) showed similar behaviour during baseline, acquisition, and reversal testing on total time spent, stimulation received, or entries made across the four corner zones within the spatial arena. **(c)** Data was pooled across eYFP pathway groups for subsequent analyses (n = 12). Data is expressed as mean ± SEM.

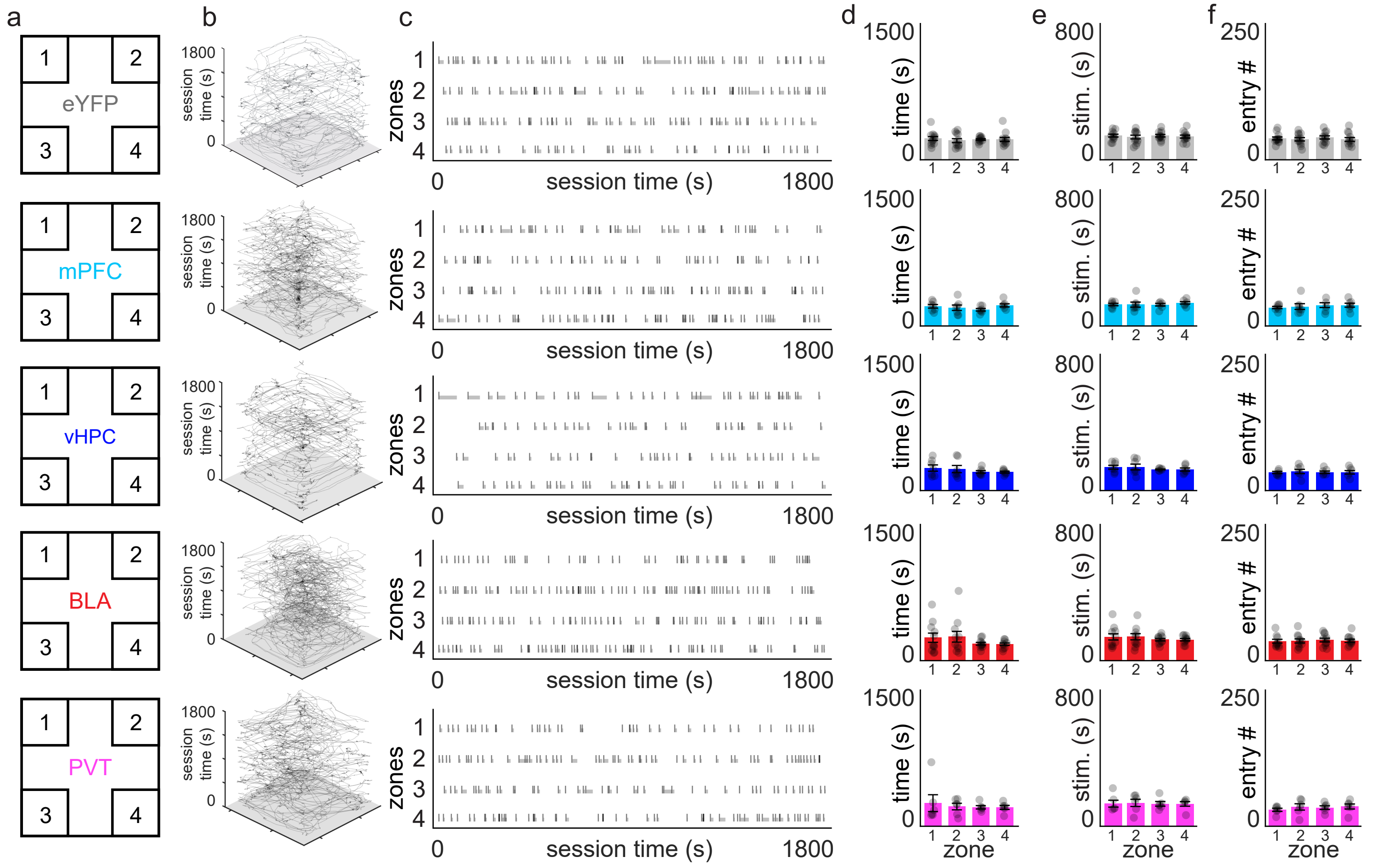

Supplementary Figure 4. No differences in baseline session exploratory behaviour in the testing arena. Different NAcSh input groups are depicted by different colours throughout: eYFP controls (grey, n = 12), mPFC→. NAcSh (cyan, n = 6), vHPC→NAcSh (blue, n = 7), BLA→NAcSh (red, n = 11), and PVT→NAcSh (pink, n = 6). **(a)** No stimulation was provided in any of the corner zones during the 30 m baseline session. **(b)** x-y position tracking for representative mice throughout the session. **(c)** Raster plots showing individual zone entries over time in representative mice (same mice as b). **(d-f)** No differences noted in any measure of maze exploratory behaviour across the four corner zones, including total time spent **(d)**, mock stimulation time (**e**; see Supplementary Fig. 2), or zone entry number **(f)** for any group during the baseline session. Data is expressed as mean ± SEM.

a

mPFC-NAcSh contingent stimulation
(acquisition session)

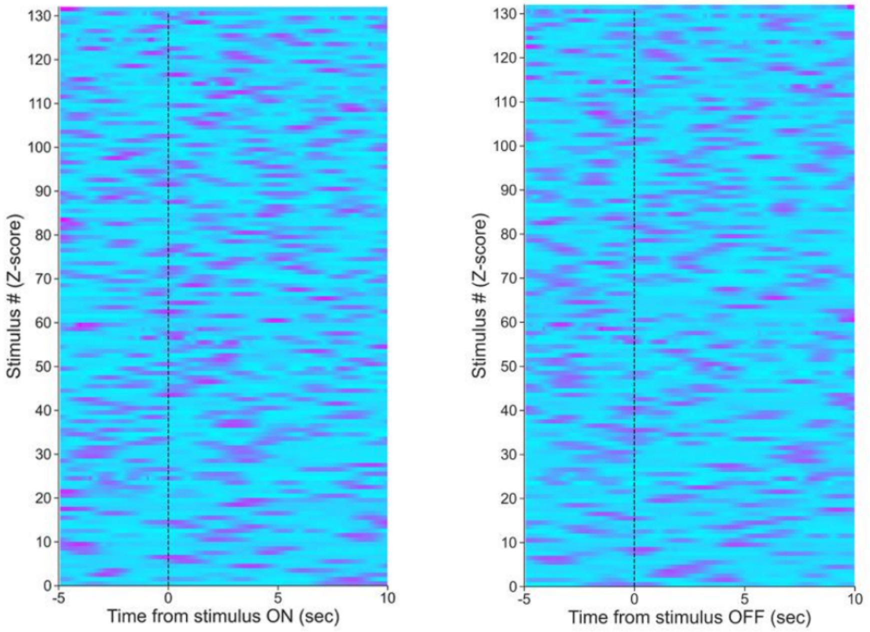

b

mPFC-NAcSh noncontingent stimulation
(ON epoch)

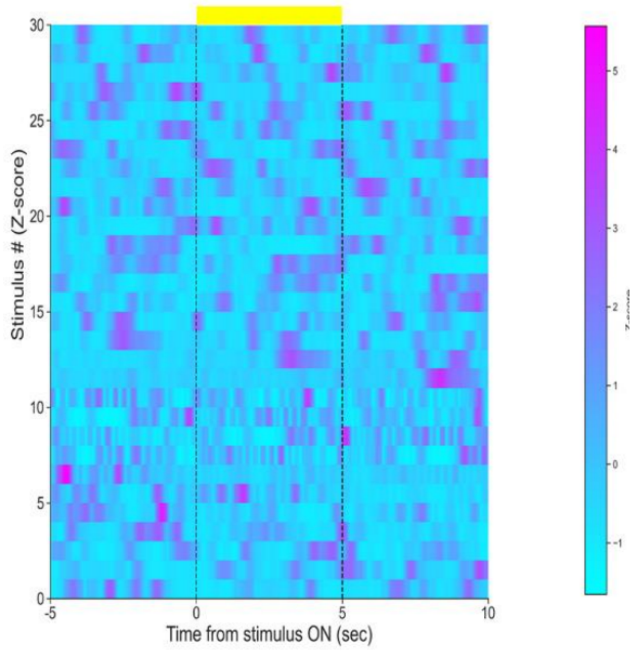

Supplementary Figure 5. No obvious velocity changes paired with stimulus onset or offset in mice receiving either contingent or non-contingent stimulation of mPFC→NAcSh inputs. Representative heat maps of velocity changes (z-scores) before and after active contingent stimulation onset **(a)** or offset **(b)** in a representative mPFC→NAcSh mouse during ongoing self-stimulation during an acquisition session. **(c)** Representative heat map of velocity changes (z-scores) before and after passive non-contingent stimulation onset and offset (yellow line = 5 sec of stimulation) during the 5 min on period.

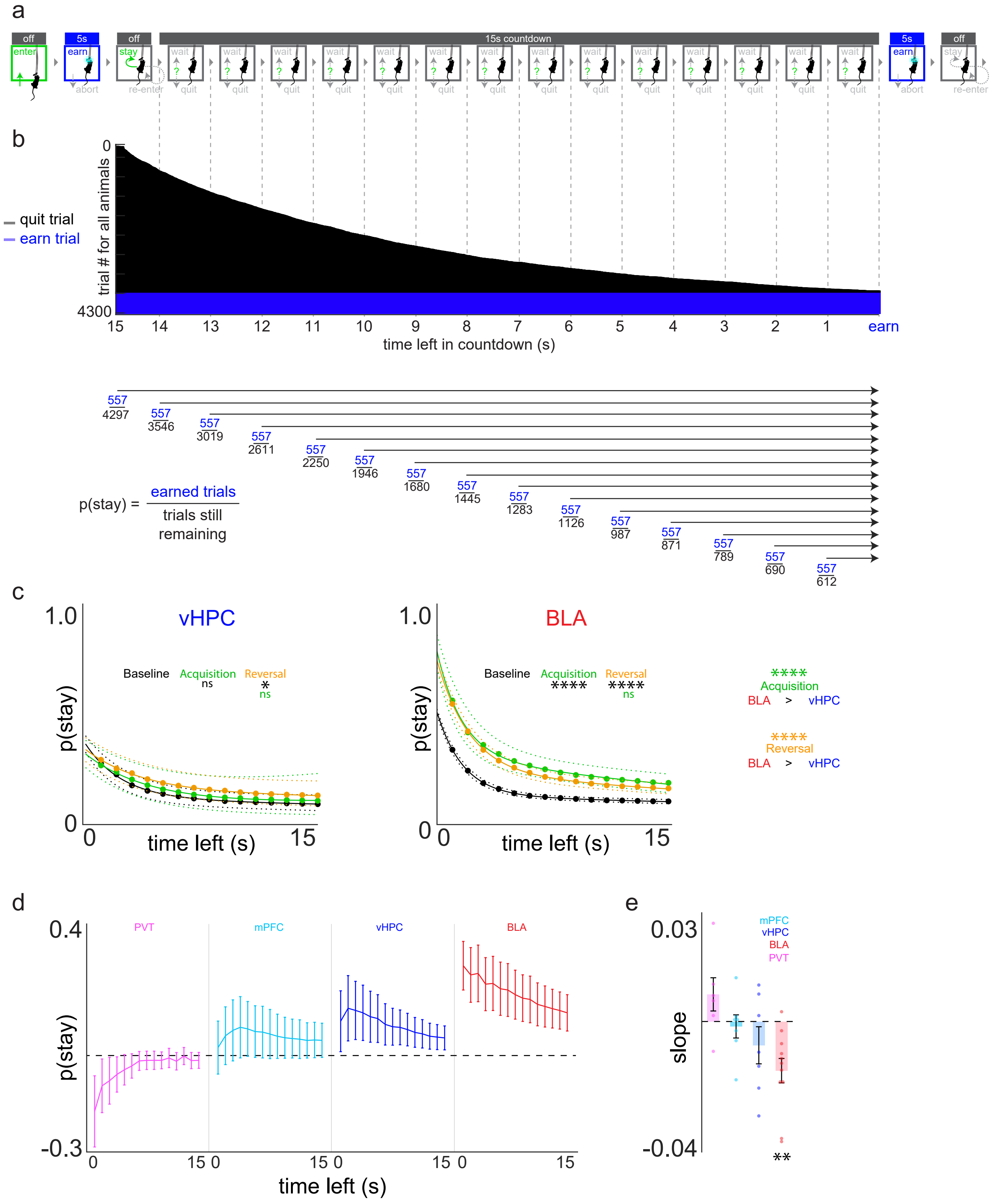

Supplementary Figure 6. BLA→NAcSh mice exhibit enhanced sensitivity to reward-related time investments. (a) Schematic showing procedure used to assess the probability to remain in the corner paired with optogenetic stimulation as a function of time already invested, (p(stay)). **(b)** Data are from all stay trials pooled across animals within a given session and/or brain region. Stay trials occurred when mice stayed in the active zone past the initial 5 sec of stimulation to 7 initiate the timeout countdown. p(stay) values were calculated at each 1 sec time bin across the 15 sec timeout period. **(c)** Sensitivity to time investments ('sunk costs') in vHPC→NAcSh and BLA→NAcSh mice. Data is expressed as mean ± SEM (dashed lines). Mixed-model ANOVA analysis indicated an overall three-way interaction between brain region x session x time left in the countdown: $F_{2,78}=3.7009$, $p=0.0291$. Tukey's post hoc comparisons revealed that sunk cost effects were apparent in BLA→NAcSh mice during both acquisition and reversal sessions compared to baseline, but only during reversal in vHPC→NAcSh mice. However, sensitivity to these 'sunk costs' were also more pronounced in BLA→NAcSh mice compared vHPC→NAcSh. **(d)** Detrended curves (mean ± SEM) showing differences in p(stay) between baseline and active (acquisition and reversal) stimulation sessions across brain regions. Data is averaged across inactive (baseline) and active (acquisition and reversal) zones. **(e)** One-way ANOVA analysis of slope values derived from detrended p(stay) curves in individual mice also show differences across NAcSh inputs ($F_{3,27} = 4.7486$, $p = 0.0087$). A sign test within each region further revealed that only BLA→NAcSh mice had altered slopes compared chance levels ($t_{10} = -4.0190$, $^{**}p = 0.0012$).

total time

		mPFC	vHPC	BLA	PVT
vs mPFC	t =		-1.04	1.14	5.66
	p =		0.9685	0.9466	**<.0001**
vs vHPC	t =			2.29	6.65
	p =			0.3188	**<.0001**
vs BLA	t =				5.11
	p =				**<.0001**

stimulation time

		mPFC	vHPC	BLA	PVT
vs mPFC	t =		2.14	5.57	7.62
	p =		0.3904	**<.0001**	**<.0001**
vs vHPC	t =			3.2	5.56
	p =			**0.0336**	**<.0001**
vs BLA	t =				3.05
	p =				**0.0512**

wait time

		mPFC	vHPC	BLA	PVT
vs mPFC	t =		-2.72	-1.83	3.08
	p =		0.1227	0.5993	**0.0476**
vs vHPC	t =			1.17	5.69
	p =			0.9385	**<.0001**
vs BLA	t =				5.12
	p =				**<.0001**

total stimulations

		mPFC	vHPC	BLA	PVT
vs mPFC	t =		3.45	5.66	6.18
	p =		**0.0152**	**<.0001**	**<.0001**
vs vHPC	t =			1.85	2.86
	p =			**0.0182**	**0.0859**
vs BLA	t =				1.38
	p =				0.8666

entry stimulation

		mPFC	vHPC	BLA	PVT
vs mPFC	t =		3.89	6.02	5.44
	p =		**0.0033**	**<.0001**	**<.0001**
vs vHPC	t =			1.71	1.7
	p =			0.6790	0.6893
vs BLA	t =				0.23
	p =				1.0000

stay stimulation

		mPFC	vHPC	BLA	PVT
vs mPFC	t =		-2.25	-2.36	1.46
	p =		0.3277	0.2648	0.1455
vs vHPC	t =			0.12	3.62
	p =			1.0000	**0.0086**
vs BLA	t =				3.85
	p =				**0.0038**

sunk costs

		mPFC	vHPC	BLA	PVT
vs mPFC	t =		-2.16	-11.29	-2.14
	p =		0.3811	**<0.0001**	0.3952
vs vHPC	t =			-9.13	0.02
	p =			**<0.0001**	1.0000
vs BLA	t =				9.15
	p =				**<0.0001**

Supplementary Figure 7. Detailed statistical analyses for direct comparison of optogenetically-induced behavioural strategies across NAcSh inputs. Following mixed model ANOVA analyses that indicated significant input x zone (pooled active and pooled inactive) interaction effects in each category in Fig. 4, Tukey's post hoc comparisons were done to compare across inputs within the active zones and yielded the values included here. $p < 0.05$ results are shown in bold.

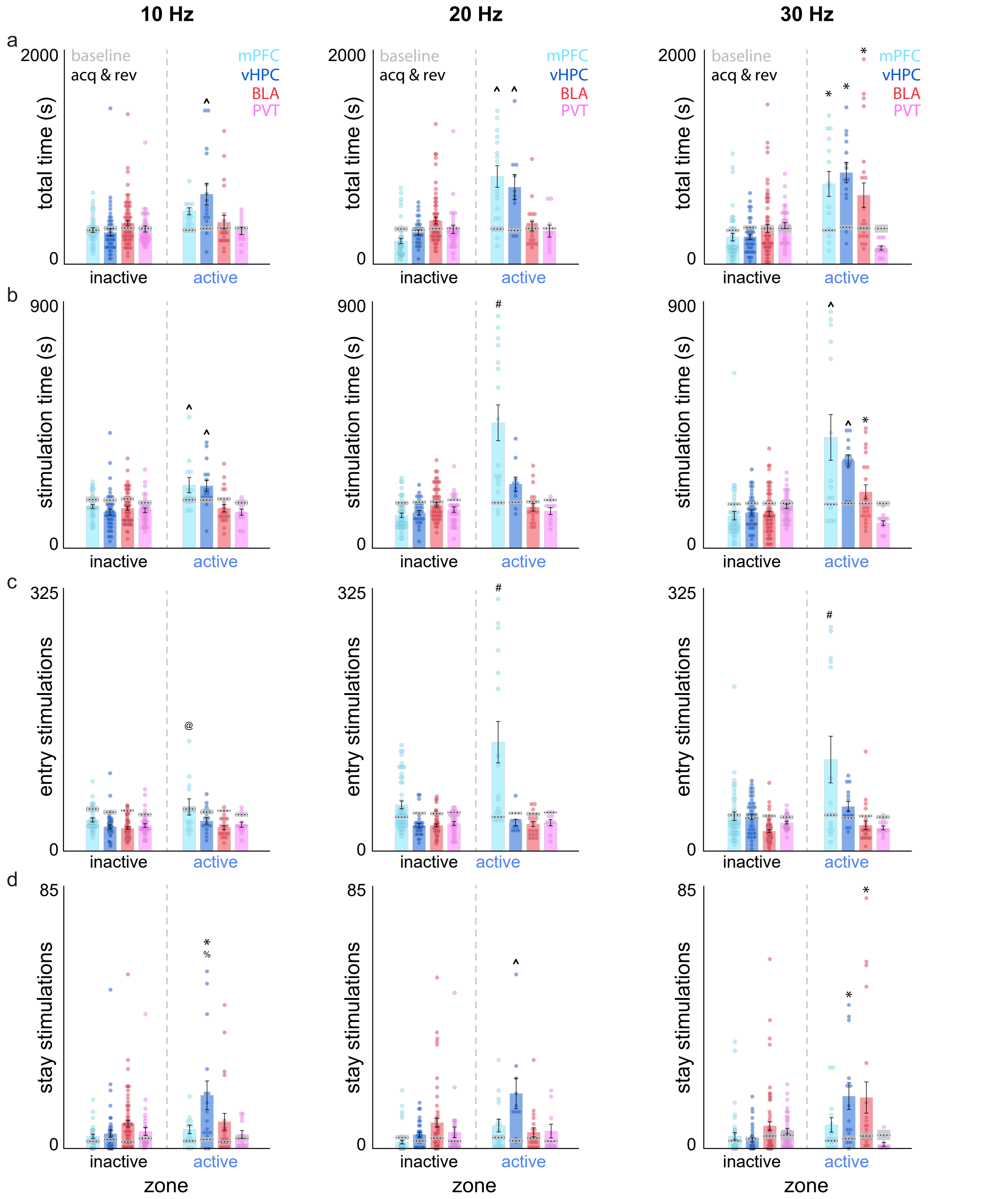

Supplementary Figure 8. Cross-pathway strategy differences are apparent in mice self-administering a range of optogenetic stimulation frequencies. Pooled data from acquisition and reversal testing is presented for both active (closed bars) and inactive (open bars) zones, with data from baseline in grey for reference. Data is expressed as mean ± SEM. Separate cohorts of mice were used to examine behaviour zones at each frequency (10, 20, or 30 Hz) and brain region (mPFC→NAcSh (cyan; 10 Hz n=6, 20 Hz n=8, 30 Hz n=7), vHPC→NAcSh (blue; 10 Hz n=8, 20 Hz n=5, 30 Hz n=7), BLA→NAcSh (red; 10 Hz n=11, 20 Hz n=12, 30 Hz n=11), and PVT→NAcSh (pink; 10 Hz n=6, 20 Hz n=5, 30 Hz n=6). **(a)** zone x brain region effects on time in zone were found at 10 Hz ($F_{3,213}$ = 7.1134, p=0.0001), 20 Hz ($F_{3,192}$ = 21.2189, p<0.0001), and 30 Hz ($F_{3,213}$ = 19.2294, p<0.0001). **(b)** zone x brain region effects on optogenetic stimulation delivered were found at 10 Hz ($F_{3,213}$ = 8.4811, p<0.0001), 20 Hz ($F_{3,192}$ = 40.1421, p<0.0001), and 30 Hz ($F_{3,213}$ = 19.2294, p<0.0001). **(c)** zone x brain region effects on entry stimulations were found at 10 Hz ($F_{3,213}$ = 3.9476, p=0.0091), 20 Hz ($F_{3,192}$ = 34.6439, p<0.0001), and 30 Hz ($F_{3,213}$ = 18.1867, p<0.0001). **(d)** zone x brain region effects on the number of stay stimulations obtained were found at 10 Hz (F3,213 = 5.2485, p=0.0016), 20 Hz ($F_{3,192}$ = 7.1277, p=0.0001), and 30 Hz ($F_{3,213}$ = 5.3269, p=0.0015). Tukey's post hoc comparisons across inputs and within active zones are indicated as follows: *p<0.05 vs. PVT→NAcSh, @p<0.05 vs. BLA→NAcSh, %p<0.05 vs. mPFC→NAcSh, ^p<0.05 vs. BLA→NAcSh and PVT→NAcSh, #p<0.05 vs. vHPC→NAcSh, BLA→NAcSh, and PVT→NAcSh, &p<0.05 vs. mPFC→NAcSh, vHPC→NAcSh, and PVT→NAcSh.
