# Supplementary material for: A quadruple dissociation of reward-related behaviour in mice across excitatory inputs to the nucleus accumbens shell
Source: Commun Biol. 2023 Jan 30;6:119. doi: 10.1038/s42003-023-04429-6 (PMC9886947; doi:10.1038/s42003-023-04429-6)
Supplement: Supplementary file 4 — Reporting Summary [file 42003_2023_4429_MOESM4_ESM.pdf]

nature portfolio

Corresponding author(s): Mark Thomas, Ph.D.

Last updated by author(s): Nov 11, 2022

Reporting Summary

Nature Portfolio wishes to improve the reproducibility of the work that we publish. This form provides structure for consistency and transparency in reporting. For further information on Nature Portfolio policies, see our Editorial Policies and the Editorial Policy Checklist.

Statistics

For all statistical analyses, confirm that the following items are present in the figure legend, table legend, main text, or Methods section.

n/a	Confirmed	
☐	☒	The exact sample size (*n*) for each experimental group/condition, given as a discrete number and unit of measurement
☐	☒	A statement on whether measurements were taken from distinct samples or whether the same sample was measured repeatedly
☐	☒	The statistical test(s) used AND whether they are one- or two-sided<br>*Only common tests should be described solely by name; describe more complex techniques in the Methods section.*
☒	☐	A description of all covariates tested
☐	☒	A description of any assumptions or corrections, such as tests of normality and adjustment for multiple comparisons
☐	☒	A full description of the statistical parameters including central tendency (e.g. means) or other basic estimates (e.g. regression coefficient) AND variation (e.g. standard deviation) or associated estimates of uncertainty (e.g. confidence intervals)
☐	☒	For null hypothesis testing, the test statistic (e.g. *F*, *t*, *r*) with confidence intervals, effect sizes, degrees of freedom and *P* value noted<br>*Give P values as exact values whenever suitable.*
☒	☐	For Bayesian analysis, information on the choice of priors and Markov chain Monte Carlo settings
☒	☐	For hierarchical and complex designs, identification of the appropriate level for tests and full reporting of outcomes
☐	☒	Estimates of effect sizes (e.g. Cohen's *d*, Pearson's *r*), indicating how they were calculated

Our web collection on statistics for biologists contains articles on many of the points above.

Software and code

Policy information about availability of computer code

Data collection	Behavioral data was collected using video cameras (Unibrain Fire-i) and ANY-maze behavior tracking software (Stoelting Co., v5.05jbeta). Histology images were taken at 4, 10, or 20x using an epifluorescent microscope (Leica Biosystems, Application Suite v4.9)
Data analysis	ANY-maze behavioral tracking software was used to measure behavioral counts across 5 min time bins or total counts across the 30 min self-stimulation sessions. ANY-maze .csv output files were used to further process data using either Matlab or Python code. Data was analyzed using JmpPro13/15 or GraphPad Prism9. One-way, two-way, two-way repeated, or mixed model ANOVAs were used to identify main and interaction effects, followed by post hoc comparisons using either Student's (for a priori comparisons) or Tukey's multiple comparisons tests.

For manuscripts utilizing custom algorithms or software that are central to the research but not yet described in published literature, software must be made available to editors and reviewers. We strongly encourage code deposition in a community repository (e.g. GitHub). See the Nature Portfolio guidelines for submitting code & software for further information.

Data

Policy information about availability of data

All manuscripts must include a data availability statement. This statement should provide the following information, where applicable:

- Accession codes, unique identifiers, or web links for publicly available datasets
- A description of any restrictions on data availability
- For clinical datasets or third party data, please ensure that the statement adheres to our policy

Source data for the figures is available in the provided Extended Data file. Raw ANY-maze .csv files and Matlab/Python code are available by reasonable request to the corresponding author.

Human research participants

Policy information about studies involving human research participants and Sex and Gender in Research.

Reporting on sex and gender	n/a
Population characteristics	n/a
Recruitment	n/a
Ethics oversight	n/a

Note that full information on the approval of the study protocol must also be provided in the manuscript.

Field-specific reporting

Please select the one below that is the best fit for your research. If you are not sure, read the appropriate sections before making your selection.

☒ Life sciences ☐ Behavioural & social sciences ☐ Ecological, evolutionary & environmental sciences

For a reference copy of the document with all sections, see nature.com/documents/nr-reporting-summary-flat.pdf

Life sciences study design

All studies must disclose on these points even when the disclosure is negative.

Sample size	No statistical tests were used to predetermine sample size. Sample sizes were selected based on typical numbers utilized for assessment of behavior in an open field apparatus.
Data exclusions	Mice were excluded for incorrect viral or optical fiber targeting.
Replication	Acquisition data of the mPFC-NAcSh group was replicated across two different experiments in independent groups of mice (i.e., acquisition/reversal testing and acquisition/extinction). Separate groups of mice were also assessed at different stimulation frequencies and similar effects were found albeit at lower levels as frequency decreased. Ongoing studies in the lab have also replicated the basic findings of these studies regarding behavioral metrics during acquisition and reversal testing.
Randomization	Animals were randomly assigned to viral targeting group, self-stimulation frequency, and behavioral apparatus being tested in (six different apparatuses were available).
Blinding	Experimenters were often, but not always, blinded to the identity of the experimental input-specific pathway groups being run for behavioral testing. However, behavioral testing was unbiased as it was scored independent of the experimenter by the ANY-maze software.

Reporting for specific materials, systems and methods

We require information from authors about some types of materials, experimental systems and methods used in many studies. Here, indicate whether each material, system or method listed is relevant to your study. If you are not sure if a list item applies to your research, read the appropriate section before selecting a response.

Materials & experimental systems

n/a	Involved in the study
☒	☐ Antibodies
☒	☐ Eukaryotic cell lines
☒	☐ Palaeontology and archaeology
☐	☒ Animals and other organisms
☒	☐ Clinical data
☒	☐ Dual use research of concern

Methods

n/a	Involved in the study
☒	☐ ChIP-seq
☒	☐ Flow cytometry
☒	☐ MRI-based neuroimaging

Animals and other research organisms

Policy information about studies involving animals; ARRIVE guidelines recommended for reporting animal research, and Sex and Gender in Research

Laboratory animals	Wild-type male C57BL/6 mice (Jackson Laboratories) aged ~6 weeks at start of study.
Wild animals	n/a
Reporting on sex	Only male mice were used in this study.
Field-collected samples	n/a
Ethics oversight	All experimental procedures were approved by the University of Minnesota Institutional Animal Care and Use Committee and followed guidelines of the American Association for the Accreditation of Laboratory Animal Care.

Note that full information on the approval of the study protocol must also be provided in the manuscript.
